# Shoulder injuries in rugby union: a systematic review and meta-analysis

**DOI:** 10.1007/s00402-026-06445-7

**Published:** 2026-07-29

**Authors:** Jack Doyle, Matthew Bellamy, Kieran Fowler, Harry Keiller, Roshan Gunasekera, Lennard Funk

**Affiliations:** 1 University of Sheffield, Sheffield, UK; 2 Sheffield Teaching Hospitals NHS Foundation Trust, Sheffield, UK; 3 The Wilmslow Hospital, HCA, Manchester, UK

**Keywords:** Sports medicine, Epidemiology, Rugby union, Shoulder instability, Sports injury, Return to play

## Abstract

Shoulder injuries are common in Rugby Union, resulting in prolonged absence and considerable burden. Reported incidence, severity, and mechanisms vary widely, reflecting the multiple high-impact actions in the sport. Various management approaches exist to treat a wide range of pathologies. This systematic review and meta-analysis aimed to synthesise evidence on the epidemiology, mechanisms, and treatment of shoulder injuries in rugby. PUBMED, SCOPUS, and WEB OF SCIENCE were searched from database inception until 1st June 2025. From 1,099 abstracts screened, 37 studies were included. The pooled match incidence was 11.02 injuries/1,000 player-hours (95% CI 7.23–16.82), though heterogeneity was very high (I² = 97%) and the 95% prediction interval was wide (2.11–57.61 injuries/1,000 player-hours), indicating the pooled rate should be read as an average across diverse populations rather than a single representative value. Higher rates were seen in male elite (13.74/1,000 h) and high school/university players (12.28/1,000 h). Training incidence was considerably lower (0.20/1,000 h). Substantial between-study heterogeneity was observed across all incidence analyses, reflecting genuine variation across playing levels, sex, and surveillance methods. Tackling was the predominant injury mechanism, most often affecting the tackler. Bankart, Latarjet, and Bristow procedures all produced favourable reported outcomes for surgical management of anterior shoulder instability. These were indirect comparisons drawn from observational cohorts, however, and comparative effectiveness between techniques cannot be determined from the available evidence. Shoulder injuries in rugby occur far more often in matches than in training, result in prolonged time loss, and are most sustained in tackling. Surgical stabilisation is associated with favourable reported outcomes, though the comparative findings should be regarded as hypothesis-generating, and high-quality comparative studies are warranted. Greater attention to prevention, improved reporting of mechanisms, and focused research on female players should be prioritised.

**Supplementary Information:**

The online version contains supplementary material available at 10.1007/s00402-026-06445-7.

## Introduction

Rugby union is a collision sport where players endure extremely high forces through different joints, lending itself to traumatic injuries [1]. Within the elite game, players are currently much larger than players in previous eras. Between 1995 and 2015, there has been an increase in the mean player body mass of 24.3% [2]. This, with increased conditioning and athletic standards, could lead to increased collision forces in the tackle. Within rugby, there are several areas where the shoulder is specifically important. The tackle technique within rugby union, often utilises an initial shoulder contact, followed by a wrap of the arm. Due to the unpredictable nature of rugby players may receive direct contact to the shoulder through falls and non-tackle collisions. There are also rugby position-specific shoulder movements such as the overhead lineout throw and a scrum engagement, typically for hookers and props, respectively. Therefore, with the importance of the shoulder in rugby and the size of the collisions, shoulder injuries are common and impactful [3]. 

There are wide-ranging and varied figures within epidemiological studies, often depending on the subgroup analysed as well as the reporting methods utilised [4]. Match shoulder injury incidence is often lower in the community game than within the elite game, which reflects other types of injuries within rugby [5]. Gender differences within rugby injury incidences do exist; however, they vary, with some suggesting similar incidence rates and some suggesting higher incidence within the men’s game [6, 7]. Match injury risks are also higher compared to training injury risks, potentially due to the more controlled and predictable nature of training and the difference in intensity of collisions [8]. Similar trends across playing standard, gender and match/training occur for both severity and burden of injuries, though shoulder-specific comparisons remain limited.

Many sport-specific actions in rugby require the use of the shoulder to perform well, and there are also a number of sport-specific actions that put the shoulder at risk of injury. The scenario in rugby that causes the most of these injuries, as reported by the current literature, is the tackle [9]. The tackle typically involves a tackler and a tackled player, however the number of tacklers can differ. A common position of injury for the shoulder within the tackle is where the shoulder goes through forced abduction and external rotation [10]. However, due to the movement of the opposing player, the movements of the shoulder in a tackle can vary dramatically. There have also been other mechanisms of injury reported, such as try scoring, and where the player takes a direct hit to the shoulder. Reporting of mechanisms has differed between studies, with different papers focusing on different mechanisms.

A range of injuries requires a range of management approaches, and these are implemented depending on a number of factors [11]. Surgery is commonly used in players with ongoing anterior instability of the glenohumeral joint. Ongoing instability can lead to frequent dislocations or subluxations, as well as damage to the labrum and other surrounding structures. These reasonably frequently require surgery to stabilise the joint, and surgery is more often required in athletes where the sport puts greater pressure on the shoulder [12]. There are a number of different types of surgical procedures intended to re-stabilise the shoulder, and often the utilisation of them comes down to factors such as glenoid bone loss as well as the surgeon’s experience [13]. Return to play times vary between surgical techniques, and are relevant to patients aiming to return before a significant sporting event such as a tournament.

The current literature varies considerably and often focuses on small subgroups of players, both within injury epidemiology and in the reporting of management and treatment outcomes. No review or meta-analysis has yet synthesised this evidence for shoulder injuries specifically. With this systematic review and meta-analysis, we therefore aim to synthesise epidemiological data, describe mechanisms of shoulder injuries within this sport, and evaluate treatment outcomes, to help inform clinical practice and injury prevention strategies.

## Methods

The systematic review and meta-analysis’ protocol was prospectively registered and published with PROSPERO (CRD420251065020). The review was conducted following the PRISMA (Preferred Reporting Items for Systematic reviews and Meta-Analyses) guidelines [14]. The review addressed three primary objectives: (1) to estimate the incidence, severity, and burden of shoulder injuries in rugby union across playing levels and sexes; (2) to describe and synthesise the mechanisms of shoulder injury in rugby union; and (3) to evaluate surgical and non-surgical treatment outcomes for anterior shoulder instability in rugby union players. The eligibility criteria were defined using a PICO framework and are summarised in Table 1.

**Table 1:** PICO Eligibility Framework

PICO	Inclusion	Exclusion
P	Male and female rugby union players of all ages and levels.	Studies where rugby union-specific shoulder injury data cannot be extracted.
I	Rugby-specific activities and preventive or therapeutic interventions.	Studies focused solely on other sports, rehabilitation or injury types.
C	Player characteristics (e.g., level, age, gender), treatment types (surgical vs. conservative), or standard practice.	Non-rugby union research (e.g., rugby league, rugby sevens) unless data for rugby union is separately extractable.
O	Shoulder injury incidence, mechanisms, risk factors, treatment outcomes, rehabilitation, and prevention effectiveness.	Case reports, reviews, editorials, commentaries, and conference abstracts without primary data.

We searched PUBMED, SCOPUS and Web of Science from the dates of their inception until the 1st of June 2025. The full search strategy for each database, including all keywords, MeSH terms, Emtree terms, Boolean operators, date restrictions, and language restrictions, is provided in Supplementary Material 1. Briefly, search terms included shoulder anatomical terms (e.g., shoulder, glenohumeral, rotator cuff, labrum, dislocation, instability), rugby-specific terms (e.g., rugby union, rugby player) and injury and epidemiology terms (e.g., injury, incidence, prevalence, surveillance). Searches were not restricted by language at the database level, though only English-language full texts were included at the full-text screening stage. Randomised controlled trials, case-control, prospective cohort, observational, correlational, cross-sectional and longitudinal studies were eligible for inclusion. Case series were eligible if a minimum of five individual cases were reported. In practice, no randomised controlled trials, case-control, cross-sectional or longitudinal studies met the eligibility criteria at full-text screening, and the final included set comprised cohort studies and case series only. The risk-of-bias tool applied to each study therefore corresponded to its true design (Newcastle-Ottawa Scale for cohort studies; JBI Critical Appraisal Checklist for Case Series for case series), and the Cochrane RoB 2.0 and MINORS tools were not required. Any systematic reviews were not included in data collection, but their bibliographies were searched for other relevant studies.

Any study that included extractable data on shoulder injuries in rugby union was included. This includes studies that include multiple sports, or rugby papers involving other types of injuries, where rugby union-related shoulder injury data could be extracted. Studies with male and female rugby union players of all ages and playing levels (elite, amateur, youth), participating in training or competition, met our study criteria. Relevant outcomes that were included in our analysis included epidemiological data, mechanisms of injury, risk factors, management and treatment outcomes. Within epidemiological factors incidence, severity and burden were assessed within different playing populations. Descriptive studies about sport-specific mechanisms of injury had their results extracted. Both intrinsic and extrinsic risk factors were included. Management and treatment outcomes had key outcomes such as return to sport times, reinjury rates and PROMs included.

###  Selection process

All titles and abstracts included were imported into the review platform ‘Rayyan’. Duplicates were removed on the Rayyan review platform prior to any screening process. Two reviewers then independently screened titles and abstracts based on the eligibility criteria. Full-text screening was conducted by the same blinded independent reviewers. Any disagreements at either stage were resolved by a third reviewer. Data extraction was performed by the principal investigator alone, with queries resolved by a second reviewer not involved in data collection. Dual independent data extraction was therefore not undertaken, and this is acknowledged as a key methodological limitation of the review. An online collaborative data collection sheet was created to record all relevant data from the studies. Studies were then searched for background characteristics, key inclusion criteria, and any relevant outcome measures found. Data that was collected was stored on a protected shared spreadsheet and securely stored on a secure university server.

### Data items

We had three key areas of focus: epidemiology, mechanisms, and management and treatment options. Within the epidemiology, our key reported outcomes were injury incidence, severity and burden. Mechanisms were reported as a percentage of injuries caused by certain dynamics or situations within the sport. Two reviewers with rugby union knowledge were utilised where the mechanisms were reported unclearly. Management and treatment options were mainly assessed through return to sport, reinjury rate and key patient reported outcome measures (PROMs) such as the Rowe score. These were chosen as these were what were expected to be most consistently reported. For mechanism studies, the method by which injury mechanism was ascertained was recorded for each study and classified as: video analysis, clinician-reported surveillance, or patient-reported recall. Where the reporting method was not explicitly stated, patient-reported recall was assumed as the most conservative classification. A standardised categorisation framework with seven categories was applied across studies: tackling, being tackled, jackling/breakdown, set play, handoffs, try scoring, direct hits and other. Where overlapping or inconsistent terminology was used across studies, category assignment was resolved by consensus between two reviewers with rugby union knowledge.

### Risk of bias (quality) assessment

Given the anticipated predominance of observational studies and a limited number of randomized controlled trials (RCTs), critical appraisal was conducted using tools appropriate to each study design. The Newcastle-Ottawa Scale (NOS) was used for cohort and case-control studies, while cross-sectional studies were assessed using a modified NOS or the AXIS tool [15]. Case series were appraised using the Joanna Briggs Institute (JBI) Critical Appraisal Checklist for Case Series [16]. For non-randomized interventional studies, the MINORS tool was applied [17]. For RCTs, though unlikely to be common in this area, any identified were appraised using the Cochrane Risk of Bias 2.0 tool [18]. Two independent reviewers performed all assessments, with discrepancies resolved through discussion or consultation with a third reviewer. Reviewers met at the start of the assessment to go through the risk of bias assessment tools. Risk of bias was displayed utilising the ‘robvis’ tool. As no randomised controlled trials or non-randomised interventional studies met the eligibility criteria, the Cochrane RoB 2.0, AXIS and MINORS tools were not applied in practice [19]. 

### Synthesis

A narrative synthesis was conducted to summarise findings across the included studies, structured around key themes: epidemiology, mechanisms of injury, management and treatment outcomes. Data was grouped by the key outcome focus. Descriptive statistics were implemented where suitable.

###  Meta-analysis

All statistical analyses were conducted using R (version 4.4.1) with the meta and metafor packages. A single-arm meta-analysis of pooled means and standard errors was performed to obtain a single summary estimate for each outcome. The metagen function was used to pool the individual study means. For incidence rate outcomes (injuries per 1,000 player-hours), log transformation was applied prior to pooling, with random-effects pooling performed on the log scale and results back-transformed to the original scale for presentation. This approach was selected as rates are bounded at zero and the Gaussian approximation can produce uninterpretable confidence intervals containing negative values when rates are low. A continuity correction of 0.5 divided by the population size was applied to studies reporting a rate of zero, allowing them to be retained in the pooled analysis. For continuous outcomes (injury severity, surgical return-to-play, and postoperative Rowe scores), pooled means and standard errors on the original scale were retained. These outcomes represent mean durations rather than event rates and, unlike incidence and burden, were not expected to produce confidence intervals crossing zero at the values observed; they were therefore retained on the original scale. Injury burden, being a rate bounded at zero, was not pooled as a continuous outcome; pooling on the untransformed scale produced a confidence interval with a non-interpretable negative lower bound, and although the log transformation used for incidence rates removed this artefact, the resulting heterogeneity was too extreme (I² = 98.4%) for a pooled estimate to be meaningful. Burden was therefore summarised narratively.

A random-effects model was chosen for all analyses, as it accounts for the assumption that the true effect size may vary across studies due to differences in study design, population, or methodology. The pooled mean and 95% confidence interval (CI) were calculated using the Hartung-Knapp (HK) method, which provides a more conservative estimate and is robust to the low number of studies often found in meta-analyses.

Heterogeneity was assessed using the I^2^ statistic, which quantifies the proportion of total variation across studies due to true heterogeneity rather than sampling error. An I^2^ value of 0% suggests no detected statistical heterogeneity, though this should be interpreted cautiously given the limited number of studies and wide confidence intervals in some analyses; clinical heterogeneity may persist regardless of the I² value, while values of 25%, 50%, and 75% are considered to represent low, moderate, and high heterogeneity, respectively. For the overall match incidence analysis, where heterogeneity was greatest, a 95% prediction interval was additionally calculated to convey the dispersion of true effects across populations rather than only the precision of the pooled mean. A leave-one-out sensitivity analysis was also conducted for this analysis, recalculating the pooled estimate and I² with each cohort removed in turn, to assess whether the pooled rate or the observed heterogeneity was driven by any individual study. The potential influence of injury definition and surveillance method on heterogeneity was additionally explored narratively, given the distribution of cohorts across these categories. A forest plot was generated for each analysis to visually represent the individual study results and the overall pooled estimate.

## Results

### Study selection

**Fig. 1:**
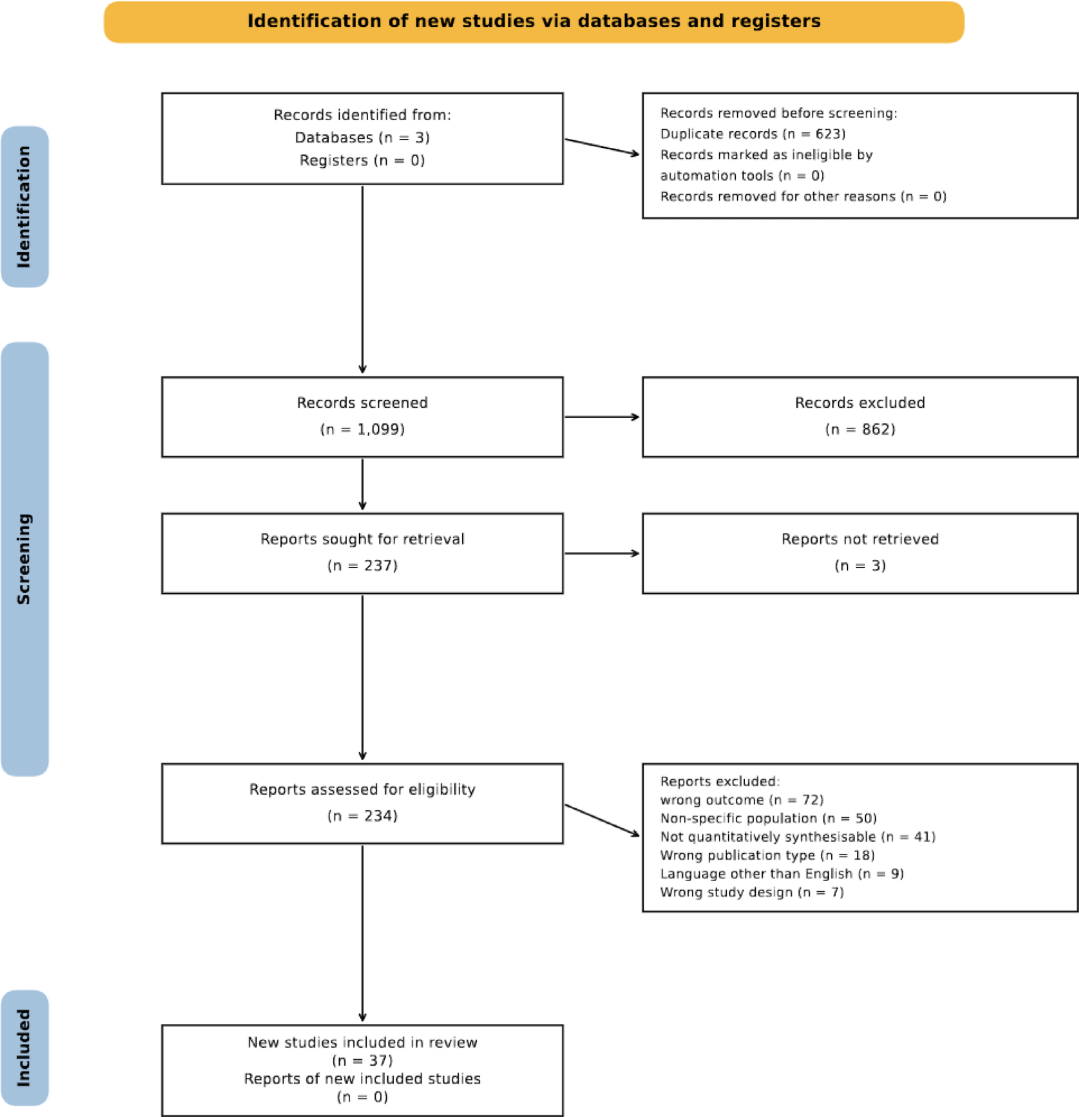
PRISMA Flow diagram

One thousand seven hundred and twenty-two abstracts were obtained, with six hundred and twenty-three of them being duplicates. After removal, one thousand and ninety-nine individual abstracts were left that fit our search criteria. After this initial abstract screening stage, it was deemed that eight hundred and sixty-two did not meet our screening criteria. Two hundred and thirty-seven full-text reports were sought for retrieval, of which two hundred and thirty-four were successfully retrieved; three could not be obtained despite multiple attempted methods of retrieval. Two hundred and thirty-four reports were therefore assessed at full-text screening. The reasons for exclusion were: wrong outcome (72); non-specific population (50); not quantitatively synthesizable (41), wrong publication type (18); studies published in a language other than English (9) and wrong study design (7). This left 37 studies included in the final review and meta-analysis [20–56] (Fig. 1).

###  Study characteristics

37 studies were able to be included in the final review (Table 2). Within these studies 33 were cohort designs, with the remaining four as case series. Publication dates ranged from 2007 to 2025. The review’s population was 10,093 players, and of these 9807 were male and 195 were female. The playing standards as reported in the studies by the authors were grouped into: elite (*n* = 15); school or university (*n* = 8); amateur (*n* = 2); mix or unstated (*n* = 11).

**Table 2:** Study demographics

Authors	Year	Study Type	Population	Age	Follow up/study duration	Male	Female	Level of playing
Van zyl	2023	Cohort	171	19.7 +/-1.7	NR	171	0	University
White	2025	Cohort	190	NR	5 seasons	0	190	Elite
Leahy	2022	Cohort	665	NR	2 seasons	665	0	Schoolboy
Leahy	2021	Cohort	665	17–19	2 seasons	665	0	Schoolboy
Murias-Lozano	2022	Cohort	258	25.3	1 season	258	0	Elite
Schwellnus	2018	Cohort	482	NR	4 season	482	0	Elite
Cruz-ferreira	2016	Cohort	51	22.1+/-4.59	1 season	51	0	Elite
Kawasaki	2014	Cohort	378	16.2+/-0.9	1 season	378	0	High-school
Usman	2014	Cohort	306	NR	4 seasons	306	0	Elite
Whitehouse	2016	Cohort	180	24.78+/-3.15	1 season	180	0	Elite
Swain	2016	Cohort	125	24.3+/-4.9	1 season	125	0	Amateur
Kawasaki	2015	Cohort	569	NR	1 season	569	0	High school and University
Schwellnus	2014	Cohort	152	NR	1 season	152	0	Elite
Leung	2017	Cohort	3585	NR	3 Seasons	3585	0	School
Barden	2018	Cohort	132	17.5+/-0.6	3 seasons	132	0	High school
Moore	2015	Cohort	78	NR	3 Seasons	78	0	Elite
Paul	2023	Cohort	94	NR	1 season	94	0	Elite
Headey	2007	Cohort	546	NR	2 seasons	546	0	Elite
Roberts	2013	Cohort	NR	NR	3 Seasons	NR	NR	Mix
Singh	2016	Cohort	NR	NR	4 seasons	NR	NR	Mix
Kawasaki	2018	Cohort	176	18.9(18.3–19.4)	48 Months	176	0	Mix
Lynch	2013	Cohort	95	25	1 season	95	0	Elite
Neyton	2012	Cohort	37	23.4	12 Years	37	0	Mix
Crichton	2012	Case Series	24	27.6	NR	24	0	Elite
Montgomery	2019	Case Series	39	25.5	Two seasons	39	0	Elite
Maki	2017	Case Series	11	25.3+/-5.9	NR	11	0	Elite
Pasqualini	2021	Cohort	88	21.3+/-4.8	59.5 months	88	0	Mix
Sundaram	2011	Cohort	184	20	3 seasons	184	0	Elite
Rossi	2021	Cohort	130	24.2	40 Months	130	0	Mix
Hirose	2023	Cohort	91	18.1+/-2.4	70+/-22.5 months	NR	NR	Mix
Tanaka	2024	Cohort	111	NR	NR	109	2	Mix
Shibuya	2021	Cohort	169	18.6	32.7 Months	169	0	Mix
Bonnevialle	2023	Cohort	62	21.6 +/-4.9	6.5 years	62	0	NR
Rossi	2020	Cohort	105	20.9+/-4.1	67.6 Months	105	0	Mix
ranalleta	2018	Case Series	49	22.8	48 months	49	0	Mix
Aso	2025	Cohort	61	18.4+/-0.5	NR	61	0	University
Hanai	2025	Cohort	34	17	48 Months	31	3	Amateur
			10,093			9807	195	

### Risk of bias in studies

**Fig. 2:**
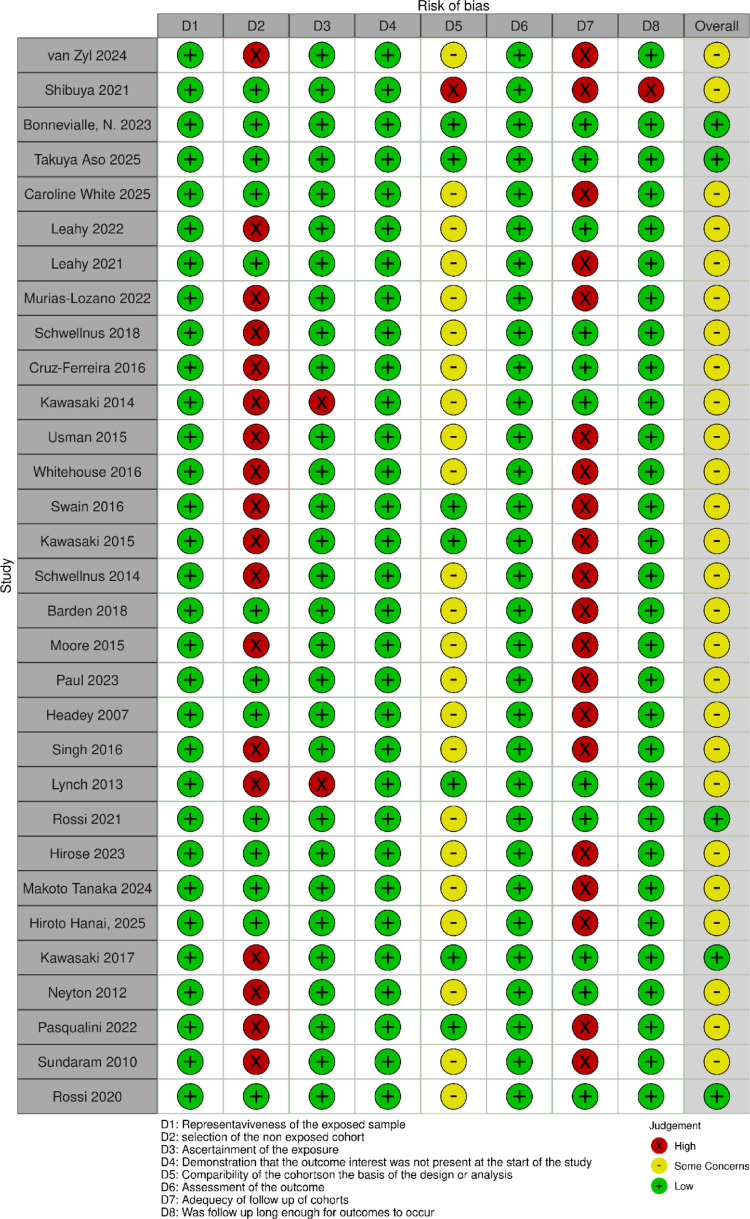
Risk of bias for the 33 included cohort studies, assessed with the Newcastle-Ottawa Scale (NOS) and visualised using the robvis tool

**Fig. 3:**
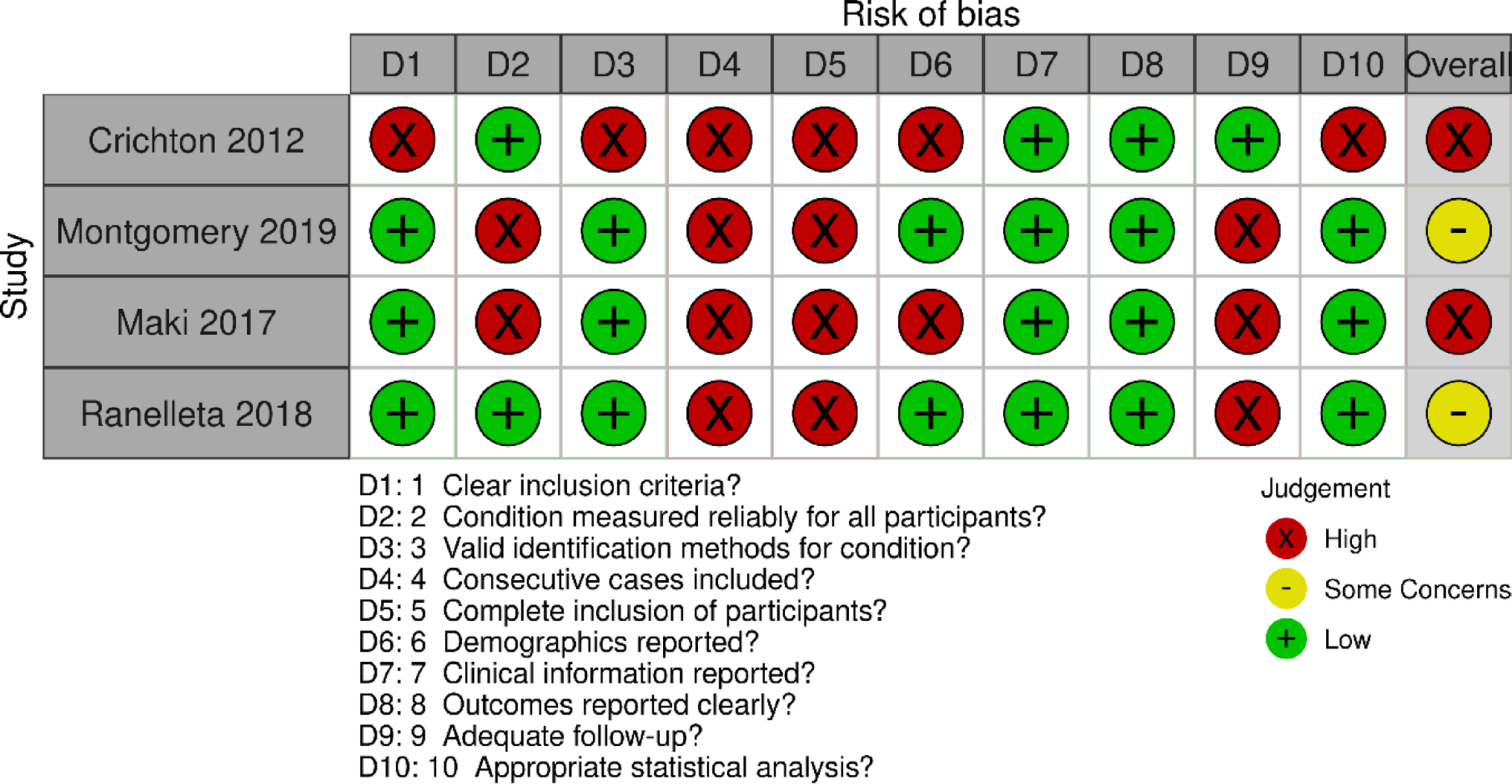
Risk of bias for the four included case series, assessed with the Joanna Briggs Institute (JBI) Critical Appraisal Checklist for Case Series and visualised using the robvis tool

Within the 33 Cohort studies (Fig. 2), our risk of bias assessment scores ranged from six to nine out of a maximum of nine, where nine out of nine reflects the lowest risk of bias. Areas of highest quality reported for the cohort studies were the representativeness of the exposed sample within the selection bias assessment. Areas of the lowest quality reported for the cohort studies were the adequacy of the follow-up of cohorts, within the outcome section of the assessment. Within the 4 case series (Fig. 3), JBI scores ranged from four to six out of a maximum of ten, where ten reflects the lowest risk of bias. Areas of highest quality reported for case series were the clear reporting of demographic and clinical information for participants (JBI items 6 and 7) and the clear reporting of outcomes or follow-up results (JBI item 8). Areas of lowest quality were the consecutive and complete inclusion of participants (JBI items 4 and 5) and the reporting of demographic information for the presenting site or clinic (JBI item 9).

### Certainty of evidence

A formal GRADE assessment was not undertaken in this review, so the judgements below reflect our overall confidence in the evidence rather than a structured certainty rating, and this is acknowledged as a limitation. Overall confidence in the evidence is low to moderate across all outcome domains, and is lowest for the surgical outcomes, where the inconsistent and incomplete reporting of recurrence, revision, complications, return to preinjury level, and glenoid bone loss precludes any assessment of comparative effectiveness. Match injury incidence had moderate certainty, with multiple prospective cohorts with reasonable consistency, limited by variability in injury definitions and exposure measurement. Training incidence had low certainty with only a few contributing studies, wide confidence intervals and statistical limitations of pooling near-zero rates. Injury severity and burden had low to moderate certainty with a few reasonable studies for severity; burden estimate was likely imprecise with a wide confidence interval. Mechanisms had low certainty with heterogeneous reporting methods, case series predominance, and retrospective ascertainment in several studies. Surgical outcomes (return to play and postoperative Rowe scores) had low certainty due to small observational cohorts, no randomised comparative studies, and significant confounding by indication. Non-operative management had very low certainty because of the single study, with there being no comparative data.

### Epidemiology

####  Injury Incidence

**Fig. 4:**
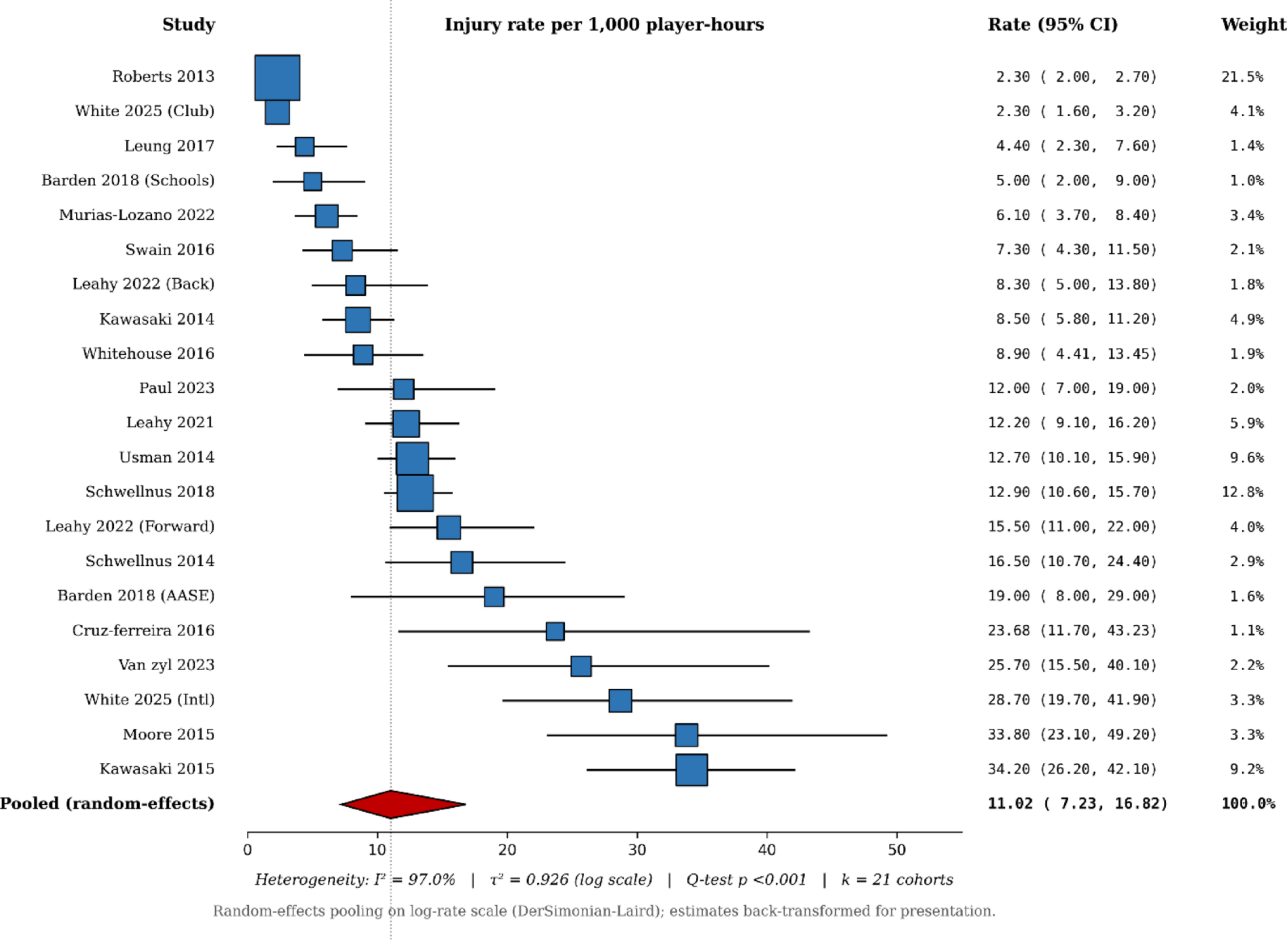
Overall Match Injury Incidence

A meta-analysis of 21 cohorts within 18 individual studies [20–36, 56] on match injury incidence found a pooled rate of 11.02 injuries (95% CI: 7.23; 16.82) per 1,000 player-hours (Fig. 4). Substantial heterogeneity was observed (I² = 97.0%, *p* < 0.001), reflecting genuine variability in shoulder injury rates across playing levels, sex, and surveillance methods. The corresponding 95% prediction interval was 2.11 to 57.61 injuries per 1,000 player-hours, indicating the range within which the true match incidence of a comparable future cohort would be expected to fall. Given the magnitude of this heterogeneity, and the width of the prediction interval relative to the confidence interval, the overall pooled rate should be interpreted as an average across diverse populations rather than a single representative value that describes any individual playing context; subgroup analyses by playing level are presented below.

 Analyses of the two largest subgroups were also conducted. In the elite male population, the meta-analysis of the eight included studies [24–26, 28, 29, 32, 35, 36] on match injury rates shows an overall pooled mean injury incidence of 13.74 injuries (95% CI: 9.97; 18.93) per 1,000 player-hours. Substantial heterogeneity was observed (I² = 84.4%, *p* < 0.001), reflecting differences in surveillance methods and league characteristics across elite cohorts. For the high school and university male level, the meta-analysis of the nine cohorts within the 7 included studies [20, 22, 23, 27, 31, 33, 34] showed an overall pooled mean injury incidence of 12.28 injuries (95% CI: 7.75; 19.44) per 1,000 player-hours. Substantial heterogeneity was again observed (I² = 91.8%, *p* < 0.001), likely reflecting variation in competition level, age group, and exposure measurement across school and university cohorts. Analysis of six studies on training injury incidence [20, 21, 27, 36, 37] revealed a pooled mean of 0.20 injuries (95% CI: 0.11; 0.34) per 1,000 player-hours. Substantial heterogeneity was observed (I² = 71.7%, *p* = 0.003), reflecting variation in playing level and training environments across cohorts.

### Sensitivity analysis

A leave-one-out sensitivity analysis showed the pooled match incidence to be robust to removal of any single cohort (range 10.39 to 12.03 injuries per 1,000 player-hours; full model 11.02), with heterogeneity remaining high throughout (I² = 92.8% to 97.2%). The lowest-rate cohort (Roberts 2013) exerted the greatest single influence, yet its removal reduced I² only to 92.8%, indicating that no individual study accounted for the heterogeneity.

Injury definition and surveillance method were also examined as potential sources. Surveillance was near-uniform (19 of 21 cohorts prospective), and restricting the analysis to the 17 time-loss cohorts did not reduce the heterogeneity (I² remained above 97%). Neither factor, therefore, explained the heterogeneity, which most plausibly reflects genuine variation in playing level, sex, competition, and exposure across populations. Subgroup analysis by sex was precluded, as only one included study reported a female cohort.

#### Injury severity

The pooled overall mean for injury severity from ten cohorts within seven studies [21, 24, 28, 29, 37, 38, 56] was 57.31 days (95% CI: 49.41; 65.21). Moderate heterogeneity was observed (I² = 48.4%), reflecting genuine variation in injury profile and follow-up across cohorts.

#### Injury burden

The meta-analysis of eight cohorts within four studies [21, 28, 34, 36] examined injury burden. Reported burden was highly heterogeneous, ranging from 2 to 1,264 days per 1,000 player-hours across cohorts (I² = 98.4%). The lowest values arose from training cohorts (2 and 39 days per 1,000 player-hours) and the highest from an elite international match cohort, with match and mixed-exposure cohorts ranging from 105 to 1,264 days per 1,000 player-hours. Given this degree of heterogeneity, and because pooling a zero-bounded rate on the untransformed scale produced a non-interpretable confidence interval, burden was not pooled into a single estimate and is presented narratively.

### Mechanisms

Nine included studies reported rugby-specific mechanisms of shoulder injury. Mechanism data were ascertained by three methods across these studies: video analysis [39, 42–44], clinician-reported surveillance [22, 40] and patient-reported recall [41, 45, 46] (Table 3). It should be noted that the patient-reported studies reported mechanism data retrospectively at the time of clinical assessment, and the video analysis studies were case series of specific dislocation events rather than prospective surveillance of all shoulder injuries, introducing potential selection bias toward more severe or well-defined injury events [23, 39–46]. Across the studies seven mechanisms were identified, being: tackling, being tackled, jackling/breakdown, set play (such as a scrum or lineout), handoffs, try scoring, direct hits, and then a non-specific ‘other’ category as well. Six of the nine studies reported the tackle as being the situation where most shoulder injuries occur [23, 39, 40, 43, 45, 46]. Other than the tackle, of the seven mechanisms reported, direct hits, try scoring, and ‘jackling’ were lesser but still significant causes of injury.

**Table 3:** Mechanism ascertainment method for the nine studies reporting rugby-specific mechanisms of shoulder injury. Studies are grouped by ascertainment method

Author	Year	Study design	Mechanism ascertainment method	*n*	References
Crichton	2012	Case series	Video analysis	24	[42]
Montgomery	2019	Case series	Video analysis	39	[43]
Maki	2017	Case series	Video analysis	11	[44]
Kawasaki	2018	Cohort	Video analysis	176	[39]
Lynch	2013	Cohort	Clinician-reported surveillance	95	[40]
Leahy	2021	Cohort	Clinician-reported surveillance	665	[22]
Neyton	2012	Case series	Patient-reported recall	37	[41]
Pasqualini	2021	Case series	Patient-reported recall	88	[45]
Sundaram	2011	Case series	Patient-reported recall	184	[46]

### Surgical management and treatment of anterior shoulder instability

The meta-analysis of 10 cohorts within 6 studies, including 775 procedures [39, 45, 47–50], looking at return to play times, found a pooled result of 5.86 months (95% CI: 5.53; 6.20) across the Latarjet, Bristow and Bankart procedures for anterior shoulder instability. No statistical heterogeneity was detected (I² = 0.0%, *p* = 0.9966), though this should be interpreted cautiously given the small number of contributing studies. Five studies were included in the pooled estimates for the Bristow procedure, with a return to play of 5.98 months (95% CI: 5.70; 6.25). Three studies were included in the pooled estimates for the Latarjet Procedure, with a return to play time of 5.23 months (95% CI: 3.84; 6.61). Two studies were included in the pooled estimates for the Bankart procedure, with a return to play time of 6.37 months. The confidence interval for this estimate (95% CI − 1.84 to 14.58) is uninterpretable, as only two studies contributed and the Hartung-Knapp adjustment on a single degree of freedom, combined with substantial between-study heterogeneity, produced an interval extending below zero. No reliable Bankart-specific return-to-play estimate can therefore be drawn, and the pooled value across all three procedures should be preferred.

**Fig. 5:**
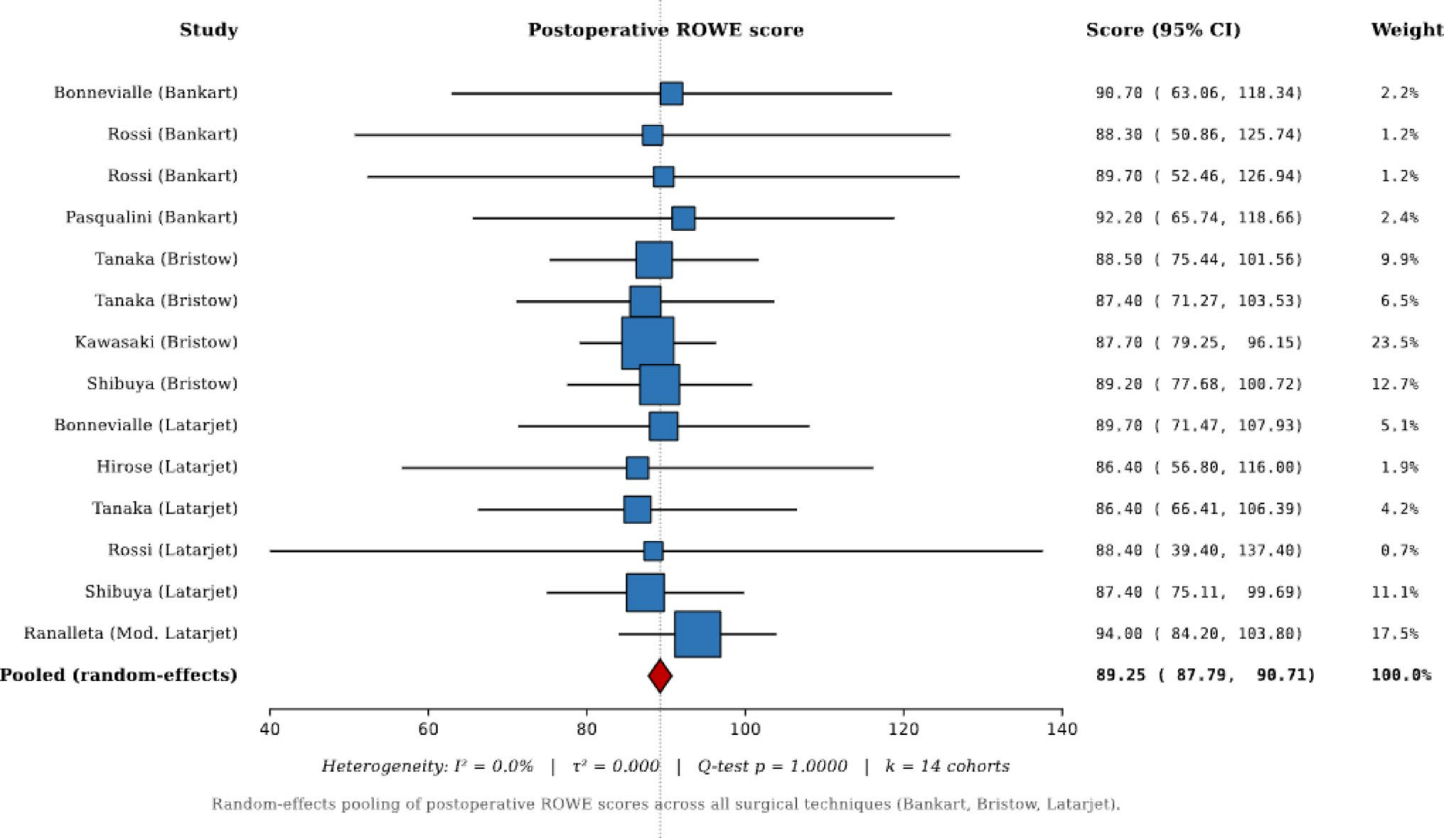
Pooled Post-Op Rowe scores

For postoperative Rowe scores, the meta-analysis of the 14 cohorts within nine studies [39, 45, 47–53] showed a pooled estimate of 89.25 (95% CI: 87.79; 90.71) (Fig. 5). No statistical heterogeneity was detected (I² = 0.0%, *p* = 1.0000), again to be interpreted cautiously given the few contributing studies. All three surgical techniques produced high pooled postoperative Rowe scores with confidence intervals that did not include zero. The pooled scores are very similar across all three groups with overlapping confidence intervals: Bankart: 90.63 (95% CI: 88.08; 93.18); Bristow: 88.17 (95% CI: 86.94; 89.40); Latarjet: 87.67 (95% CI: 86.04; 89.30).

The Bankart procedure has the highest pooled score, but the confidence intervals for all three groups overlap considerably, suggesting that there may not be a clinically significant difference between the three surgical techniques despite the small variations in their pooled estimates. It is important to note that these are indirect, non-randomised comparisons; surgical technique selection was influenced by clinical factors including glenoid bone loss, instability pattern, and surgeon preference, and these findings should therefore not be interpreted as evidence of comparative effectiveness. Preoperative Rowe scores and change scores were not consistently reported across included studies, limiting the interpretation of postoperative outcomes. Recurrence, revision, complication rates, return to preinjury level, and glenoid bone loss thresholds were reported inconsistently and incompletely across the included studies, and could not be pooled or summarised meaningfully. Given that these outcomes are central to surgical decision-making, their poor reporting is a key limitation of the surgical evidence base, and postoperative Rowe scores and return-to-play time alone are insufficient to guide procedure selection. Where available, preoperative values, recurrence rates, revision rates, and complications are described in the individual study summaries.

The key similarity is the overall lack of statistical heterogeneity (I² = 0.0% and p-values close to 1.0) in all three subgroups. While the low I² suggests limited statistical heterogeneity within subgroups, this should be interpreted cautiously given the small number of studies, wide confidence intervals in some analyses, and the inherent clinical and methodological diversity across included cohorts. Absence of statistical heterogeneity does not preclude the presence of important clinical heterogeneity.

Only one study focused on the non-surgical treatment of shoulder dislocations. Hanai et al. compared types of immobilisation for management of these players, and found that immobilisation in external rotation, alongside early muscular training, may be an effective treatment for shoulder dislocations during the in-season period [55]. 

### Risk factors

One study that was included specifically addressed and identified risk factors within rugby union [54]. They identified that ‘reduced upper thoracic mobility’ and ‘reduced scapular upward rotation angles’ were risk factors for traumatic shoulder dislocation, within a population of 61 rugby players.

## Discussion

### Research summary

Shoulder injuries in rugby are common. This meta-analysis found a pooled match incidence rate of 11.02 injuries/1,000 player-hours, with subgroup estimates of 12.28 and 13.74 injuries/1,000 player-hours for school/university and elite male populations, respectively. Training injury incidence rates were considerably lower, with our meta-analysis finding a pooled result of 0.20 injuries/1,000 player-hours. Substantial between-study heterogeneity was observed across all incidence analyses, reflecting genuine variation in playing level, sex, and surveillance methods, and the pooled rates should therefore be interpreted as averages across diverse populations. Severity varied by injury type. Injury burden was highly heterogeneous across cohorts (ranging from 2 to 1,264 days/1,000 player-hours) and was not pooled into a single estimate. The tackle was the scenario that most frequently caused shoulder injuries, with six out of nine studies reporting this finding. Surgical approach to treating anterior instability was reasonably effective across techniques, with no clear difference demonstrated in postoperative Rowe scores as well as return to play times between the different groups. Pooled return to play times for anterior instability shoulder surgery were 5.9 months, with pooled postoperative Rowe scores of 89.25 at a minimum of two years follow-up.

### Interpretation of results

The incidence of injuries in rugby union is high, reported as high as 91 injuries/1,000 player-hours in a meta-analysis by Williams et al. [57] Our meta-analysis echoed this, with a comparable shoulder-specific injury incidence of 13.74/1,000 player-hours within the elite male population. Our overall match injury incidence rate was 11.02/1,000 player-hours, reflecting the average across diverse populations and surveillance methods rather than a single representative value [21]. When subgroups were analysed for male elite as well as school and university, this gave us results of 13.74 and 12.28 injuries/1,000 player-hours respectively. These results were similar to work done on other joints within rugby union. West et al. reported a match knee injury incidence within the professional male population of 9.8/1,000 player-hours, similar to our 13.74 [58]. We were only able to include two women’s rugby cohorts, both from the same study by White et al., where they reported match incidence rates of 2.3 and 28.7/1,000 player-hours for club matches and international matches, respectively [21]. The intensity and demands of international rugby may contribute to the differences here; however, unfortunately due to the lack of studies reporting female data, we were not able to support this with any further analysis. White et al. also reported significantly lower incidence rates within the international cohort, in regard to their training hours. This was also reflected by our training injury rate of 0.20/1,000 player-hours, just under 70 times as small. This may reflect the more controllable and modifiable nature of the training environment [8]. Medical and performance teams can change the load players go under, as well as types of training and amount of contact they endure. Therefore, protecting players most at risk of injury. Our severity meta-analysis was limited by the variety of data reported and the variety of injuries focused on. There were various definitions of what constitutes a shoulder injury, such as scapula fractures. However, our pooled result of 57 days suggests a reasonable severity compared to overall injury severities within rugby which are significantly less [59]. Shoulder injury burden was highly heterogeneous across the included cohorts and could not be meaningfully pooled; reported values ranged from 2 to 1,264 days per 1,000 player-hours, driven largely by differences in exposure type and playing level, so burden is best interpreted at the level of individual populations rather than as a single pooled figure. Across all our epidemiological analyses, there may be difficulty in ascertaining true exposure hours, therefore potentially impacting the injury incidence rates.

The tackle was frequently reported as the predominant area where these shoulder injuries occurred, with the tackler being more likely to be injured than the tackled player. This reflects the literature across all types of injury, where the tackle is the highest injury-producing situation [60, 61]. However, what the majority of studies did not address was the type of tackles, with a variety of techniques and tackle situations employing different types of shoulder biomechanics, thus putting the shoulder at different levels of risk. Arm tackles displayed the greatest number of shoulder injuries in a systematic review, compared to direct head in front or direct shoulder tackles [62]. This was suggested to be down to an arm tackle displaying large shoulder abduction angles, compared to other tackles. Studies also frequently reported other mechanisms, such as try scoring and direct hits as risky scenarios for shoulder injuries. Some studies also reported the action of ‘Jackling’ or ‘poaching’ as a significant cause of shoulder injuries. Through rule changes and the progression of the game, the ‘jackal’ position, where the ball is attempted to be stripped away from the attacking team in the ruck, has become prominent. A direct link between this trend and shoulder injury rates remains a hypothesis warranting further investigation [63]. Often, the methods of reporting of mechanisms were varied, whether it be video analysis of injuries, providing potentially a more reliable view of the mechanism, or self-reporting of methods. There have not been any investigations into the effect of poor technique when completing mechanisms such as tackling, and the potential impact of fatigue. Research has shown that tackling technique worsens under fatigue within rugby union, and the impact of this on injury rates would be pertinent in creating effective injury prevention programs within training [64]. 

Surgical management was most frequently reported for anterior shoulder instability, and related pathology. Due to the frequent collisions and stresses put on the shoulder through rugby, any weakness or tendency for their shoulder to be unstable, there is often a definitive aim to surgically address this. Our included studies focused on the return to play time, as well as the most frequently reported patient-reported outcome measure (PROM) the Rowe score. We found reasonable results across all the techniques utilised, both in return to play times and with the postoperative Rowe scores. This was across all surgical techniques analysed: Latarjet, Bristow and Bankart. We also found no clear difference between the different techniques. However, the comparisons made between cohorts were limited, due to the heterogeneity of the populations. A key factor that is considered when choosing an operation technique is the status of the glenoid, often measured through the glenoid index with glenoid bone loss impacting the index score. These scores are often utilised in surgical decision-making [65]. We found varying reports of glenoid bone loss throughout the different studies, with some only implementing certain procedures if a bone loss threshold was met. There was a lack of non-operative management research, with one study looking at in-season management of anterior shoulder dislocations [55]. They found that externally rotated immobilisation, as well as early muscular training, is a useful management tool for within-season. This may allow accelerated return to play, which may be useful in the elite populations. Potentially in-season injuries in the elite population may be managed symptomatically, with players reasonably frequently managing the demands of elite rugby until the end of the season [66]. 

### Strengths and limitations

The risk-of-bias assessment had limited impact on the interpretation of most findings. For epidemiology and mechanism outcomes, the predominantly prospective surveillance design of contributing cohorts mitigates the main concern around follow-up adequacy. The limitation of incomplete follow-up has greater relevance for severity and burden estimates, as players not followed through to full return to play may have had their time-loss truncated and therefore underestimated. The greatest impact of risk of bias falls on the surgical findings, where the case series design, retrospective sampling, and inconsistent reporting of consecutive patient inclusion and follow-up adequacy substantially limit confidence in the pooled return-to-play and Rowe outcomes.

There was significant variation in the standards and techniques of reporting throughout this review and meta-analysis. There, for example, were differences in the way epidemiological data was presented. Injury incidence within sport can be represented in several ways, typically through injuries per 1,000 player-hours. However, some studies were unable to be included within the meta-analysis due to the fact that they reported injuries per 1,000 athletic exposures [67]. This therefore would not be able to be accurately compared with other studies reporting more traditionally. Injuries per 1,000 player-hours pose their own challenges, as accurately recording playing time can be difficult, especially when not in the elite professional environment [68]. Therefore, the incidence rates can be impacted, and may be inaccurately estimate injury rates across different studies, particularly in the amateur game. Shoulder injury definitions also varied between papers, and areas of focus were also not consistent. The heterogeneity in mechanism reporting methods across included studies — spanning video analysis, clinician-reported surveillance, and retrospective patient recall — limits the comparability of mechanism data and should be considered when interpreting the relative frequency of reported mechanisms. In particular, the video analysis studies were case series of dislocations rather than prospective surveillance of all injuries, and the patient-reported studies were primarily surgical cohorts where mechanisms were ascertained retrospectively, both of which may introduce selection bias. The majority of the included studies were also observational in design, with a lack of randomised controlled comparative trials. This was potentially due to the reasonably niche population that is rugby union players, and therefore study centres lacked patient numbers. However, our results do reflect comparative studies done in the general population on surgical treatment for anterior shoulder instability [69, 70]. 

We were also only able to identify two female-specific cohorts, both from within the same study, that observed shoulder injury epidemiology data within this population [21]. This prevented us from conducting meaningful sex-specific meta-analysis. We were also unable to identify any studies including amateur women’s rugby, leaving unknowns within this population. Given the very limited female-specific data available, any observations regarding sex-based differences should be considered as evidence gaps rather than conclusions, and no sex-specific generalisations should be drawn from this review. Studies within our management and treatment section often included small, single-team or single-surgeon cohorts. The significant variation in surgical decision-making also restricted the cross-study comparability between some studies. The most important limitation of the surgical evidence base, however, is the incomplete and inconsistent reporting of the outcomes that matter most to surgical decision-making. Recurrence of instability, revision surgery, complication rates, return to preinjury level of play, return-to-play rate as distinct from return-to-play time, and glenoid bone loss thresholds were reported sporadically, defined inconsistently, or omitted entirely across the included studies. None of these outcomes were reported with sufficient consistency to permit pooling, and in most cases they could not be summarised narratively in a way that would allow meaningful comparison between techniques. This is a critical gap rather than a peripheral one. Recurrence and revision are the outcomes against which stabilisation procedures are ordinarily judged, and glenoid bone loss is among the principal determinants of procedure selection; their absence means that the postoperative Rowe scores and return-to-play times synthesised here, while favourable, capture only a narrow slice of surgical performance and are insufficient on their own to guide the choice of procedure for an individual player. The incomplete reporting of preoperative PROMs and of change scores compounds this, and additionally led to some studies not reaching our threshold for analysis. Readers should therefore not interpret the favourable Rowe scores reported in this review as evidence that these procedures perform equivalently, or as a basis for procedure selection. Consistent reporting of a core outcome set for shoulder stabilisation in athletes, encompassing recurrence, revision, complications, return to preinjury level, and bone loss, is needed before comparative effectiveness in this population can be meaningfully assessed. The difficulty of inadequate reporting was also apparent through the large number of studies we had to exclude due to insufficient data to allow us to meta-analyse, such as 95% confidence intervals or standard deviations.

Our systematic review and meta-analysis, however, comprehensively searched the current literature, allowing us to analyse in depth a wide scope of studies. Our synthesis of 37 studies included over 10,000 players, over double the number of patients than the previous review done in this field [1]. We also likely implemented a broader search strategy, being able to assess nearly 1,100 individual abstracts, compared to the previous reviews of 98. We prospectively registered our protocol with PROSPERO, and followed PRISMA guidelines throughout to ensure transparency and reduce the risk of selective reporting. Although limited in our inclusion of studies focusing on women’s rugby, we were able to include this population in this systematic review for the first time for this injury type. We were able to include studies focusing on both amateur and professional rugby, as well as from youth rugby up to senior levels. Statistically, our chosen methods within the pooled analyses were well chosen to reflect the small population sizes of the subgroups, and owing to the likelihood that the populations results to differ [71]. Across our pooled results analyses, we openly acknowledged the substantial heterogeneity within the incidence analyses, and explored this through subgroup analyses by playing level rather than masking it with an inappropriate statistical approach. Through contextual knowledge of rugby, as well as post-analysis review, we were also able to map out the incidences for different populations, such as university and elite, to help accurately clarify incidence rates in specific populations.

### Implications

The findings of this review differ considerably in the strength of evidence underpinning them, and the implications below are therefore separated into those that are reasonably well supported and those that remain descriptive or hypothesis-generating. Of the findings that are reasonably well supported, the review confirms that the tackle plays the most significant role in shoulder injuries occurring in rugby union, drawing on consistent reporting across the majority of mechanism studies, and this supports technique-based prevention strategies. By contrast, the identification of risk factors rests on a single study of 61 university players, and while thoracic mobility and scapular upward rotation screening may prove useful, particularly in the elite environment, this should be regarded as a hypothesis requiring prospective validation rather than as a basis for current screening practice [54]. The markedly lower incidence of shoulder injuries in training relative to matches was consistent across the contributing cohorts, and offers some reassurance to coaching staff that controlled contact training does not appear to carry the same risk as match play, though this rests on six studies with moderate heterogeneity and the content of training was not characterised in a way that permits specific recommendations. Turning to findings that remain descriptive, our review found broadly similar reported return to play times and postoperative Rowe scores across the three surgical techniques analysed. It would be remiss not to acknowledge, however, that these estimates cannot be used to guide procedure selection. The multifactorial nature of surgical decision-making means that surgeons and athletes will continue to individualise the choice of technique to the patient, and the absence of an observed difference in our pooled estimates should not be read as evidence that the techniques are interchangeable. Surgical selection was influenced by factors including glenoid bone loss, instability pattern, and surgeon preference [72]. Our consistently high postoperative Rowe scores, across techniques, are consistent with surgical stabilisation being an effective option for athletes requiring shoulder stability for sports such as rugby, though this observation derives from uncontrolled cohorts without non-operative comparison. The suggestion that in-season patients may benefit from non-operative immobilisation in external rotation derives from a single study with no comparative arm, and is best regarded as a direction for future research rather than a clinical recommendation; further work would be required to weigh the risks against the benefits in the elite population [55]. 

Further research conducted within this field should follow standardised reporting, to help allow accurate further comparisons in the future. This is particularly highlighted within our epidemiological section, where variations in reporting prevented further studies from being included in our meta-analysis. Future research should adhere to the World Rugby consensus definitions for surveillance research [73]. Within the reporting of injury mechanisms there should be increased video analysis of injuries, particularly within the danger area of the tackle. The difference between tackle types, the effect of fatigue on technique and therefore injury rates as well as the influence of player mass, could be further assessed to help identify areas of risk. Standardised reporting is equally pressing within the surgical literature, where the inconsistent reporting of recurrence, revision, complications, return to preinjury level, and glenoid bone loss was the single greatest barrier to synthesis in this review; adoption of a core outcome set encompassing these variables should be considered a priority if the comparative effectiveness of stabilisation procedures in rugby players is to be established. Prospective multi-centred, or multi-club, cohort studies reporting such a core outcome set could more accurately assess the benefits or risks of certain surgical techniques to players. Matching the cohorts, not only by standard metrics such as age, but also by anatomical variations such as glenoid bone loss would also be beneficial. Longitudinal studies following players after injury, for a significant follow-up, would also help to identify risks of reinjury as well as long-term complications. There should be an assessment of shoulder-specific long-term impacts on players’ quality of life. Studies have found that retired elite players endure significant pain and impairment long into their retirement and understanding whether certain management strategies contribute to this more than others would help aid current decision making [74]. Understudied populations, such as female rugby union players, particularly in the community game, need studies that will assess shoulder injury risk for them. More work should be done to address positional differences, such as for front row players with specific shoulder loads related to the scrum.

Finally, considering the prevention of these injuries, or the minimisation of them, through rule changes as well as coaching changes could help to improve the safety of the sport. Progressive contact exposure, with technique reinforcement, could help to prepare players for contact in a safer environment. The emergence of shoulder injuries related to ‘jackling’ or ‘poaching’ should warrant extra surveillance. Influences such as speed of entry and body position, from the player hitting the Jackling player, could contribute to injuries. These should be further assessed, and if deemed of a significant risk rule changes should be implemented. We have seen rule changes minimising injuries, and potential injury-causing impacts, in other body regions such as minimising head contacts through tackle height rule changes [75]. So if deemed necessary by further research, these could be implemented.

###  Conclusion

This systematic review and meta-analysis demonstrate that shoulder injuries are a substantial burden within rugby union, with high match incidence rates and significant time loss across all levels of competition. The tackle remains the dominant mechanism of injury, however caution around the changing dynamics of rugby should be taken to assess mechanisms such as try scoring and ‘jackling’. Surgical management of anterior shoulder instability produced favourable reported return to play times and postoperative patient-reported outcomes across all three techniques; these were indirect comparisons from observational cohorts, however, and should be regarded as hypothesis-generating rather than as evidence that the procedures are equivalent. The evidence for non-operative management remains limited, but may be considered for elite rugby players in season. Overall, the certainty of evidence underpinning these conclusions is low to moderate, and findings should be interpreted in light of the predominantly observational study designs, methodological heterogeneity, and limitations in reporting identified across included studies.

Despite the variability in reporting standards, and the limited studies focusing on female and young athletes, this review provides the most comprehensive analysis to date on the epidemiology, mechanisms and management of rugby union shoulder injuries. Continued efforts to refine coaching and rule changes, enhance surgical management decision-making and standardise research practices will be essential to reducing the burden of shoulder injuries and improving athlete welfare across all levels of rugby.

## Supplementary Information

Below is the link to the electronic supplementary material.

Supplementary Material 1

## Data Availability

The collected data for pooled estimates and meta-analysis will be made available upon reasonable request to the corresponding author.
