# Supplementary material for: Shoulder injuries in rugby union: a systematic review and meta-analysis
Source: Arch Orthop Trauma Surg. 2026 Jul 29;146(1):273. doi: 10.1007/s00402-026-06445-7 (PMC13421216; doi:10.1007/s00402-026-06445-7)
Supplement: Supplementary file 1 — Supplementary Material 1 [file 402_2026_6445_MOESM1_ESM.pdf]

Search Strategy

(("Shoulder Injuries"[Mesh] OR "Upper Extremity"[Mesh] OR "Arm Injuries"[Mesh] OR "Clavicle"[Mesh] OR "Scapula"[Mesh] OR "Rotator Cuff"[Mesh] OR shoulder[tiab] OR upper limb[tiab] OR arm[tiab] OR clavicle[tiab] OR scapula[tiab] OR humerus[tiab] OR rotator cuff[tiab] OR glenohumeral[tiab] OR acromioclavicular[tiab] OR labrum[tiab] OR SLAP[tiab] OR upper extremity[tiab] OR Instability[tiab] OR dislocation[tiab](s) OR subluxation[tiab](s) OR Pectoralis Major[tiab] OR cartilage[tiab] OR chondral[tiab]))

AND

("Rugby"[Mesh] OR rugby[tiab] OR "rugby union"[tiab] OR "rugby league"[tiab] OR "rugby player"[tiab] OR "rugby players"[tiab])

AND

("Athletic Injuries"[Mesh] OR "Sports"[Mesh] OR "Epidemiology"[Subheading] OR injury[tiab] OR injuries[tiab] OR trauma[tiab] OR risk[tiab] OR incidence[tiab] OR prevalence[tiab] OR epidemiology[tiab] OR surveillance[tiab])
